# Subglottic stenosis due to an unexpected foreign body diagnosed after emergency tracheostomy in a child

**DOI:** 10.1186/s40981-019-0268-4

**Published:** 2019-07-17

**Authors:** Tatsuya Tsuji, MinHye So, Kazuya Sobue

**Affiliations:** Department of Anesthesiology and Intensive Care Medicine, Graduate School of Medical Sciences, Nagoya City University, 1 Kawasumi, Mizuho-cho, Mizuho-ku, Nagoya, Japan

**Keywords:** Subglottic stenosis, Emergent tracheostomy, Child, An unexpected foreign body

## Abstract

**Background:**

Subglottic stenosis is a known complication of traumatic and prolonged endotracheal intubation. It is rare that the causes of severe subglottic stenosis are revealed to be an unexpected foreign body after airway securement in a child. Subglottic stenosis in a child is often associated with airway emergency, and management of difficult airway may be required.

**Case presentation:**

We report the case of an 8-year-old girl with severe subglottic stenosis who required emergency tracheostomy. Emergency tracheostomy was performed under regional anesthesia. Sevoflurane was administered with sufficient titration to maintain spontaneous breathing. At first, the cause of severe subglottic stenosis was thought to be a traumatic event that had occurred 1 month previously; however, subsequent laryngoscopy revealed that the cause of subglottic stenosis was a foreign body.

**Conclusions:**

Management of the airway in a child with severe subglottic stenosis should be selected according to each patient’s individual circumstances.

## Background

The most common cause of acquired subglottic stenosis is trauma, which can be internal (prolonged endotracheal intubation, tracheostomy, flame burn injury) or external (blunt or penetrating neck trauma) [1]. It is rare for severe subglottic stenosis to be caused by an unexpected foreign body after airway securement following an external traumatic event in a child. Acquired subglottic stenosis in a child can be a life-threatening situation requiring immediate airway management. However, many practice guidelines for management of the difficult airway primarily focus on the upper airway and thus may not be helpful in such a case. We experienced difficult airway management in a child with severe subglottic stenosis.

## Case presentation

An 8-year-old girl arrived at the emergency department with severe respiratory obstruction following interhospital transport. She weighed 42 kg and was 137 cm in height. Treatment for asthma that had been initiated about 1 month before had not improved her respiratory condition. She presented with marked stridor and cough, severe intercostal and subcostal chest retraction, and developed acute respiratory distress. Her parents mentioned that her respiratory condition might have been worsened by an external traumatic event at the neck that had been caused by her younger brother a few months earlier. At that time, while she had been playing with her younger brother, his elbow had bumped into her neck violently.

**Fig. 1:**
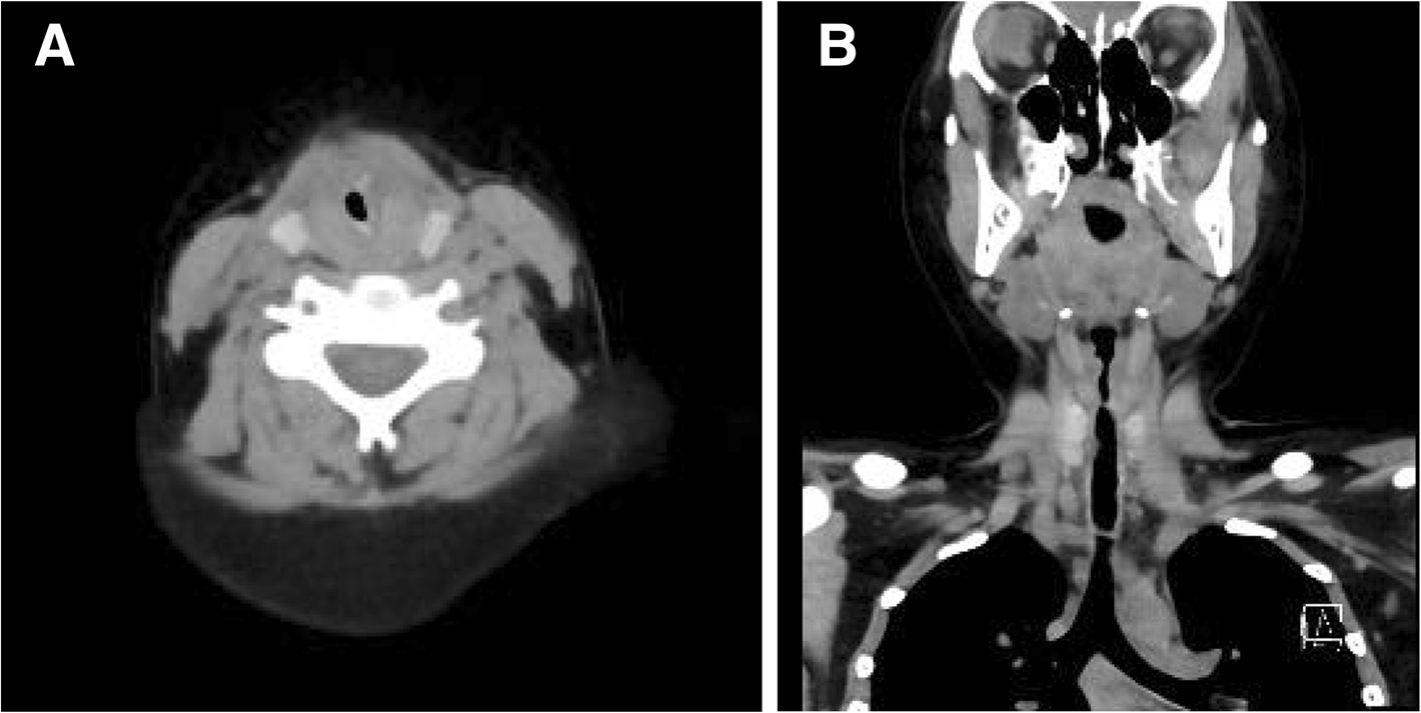
CT images of the neck with severe subglottic stenosis on admission

On admission, computed tomography (CT) of the neck showed that severe subglottic stenosis has developed with minimal cross-sectional diameters of 3.5 × 1.8 mm at the narrowest point (0.05 cm^2^) (Fig. 1). Laryngoscopy revealed that her airway was almost completely obstructed by severe subglottic stenosis. Findings from other preoperative investigations including laboratory tests, chest X-ray, and electrocardiography were normal. The preoperative vital signs were blood pressure of 120/59 mmHg, temperature of 37.5 °C, respiratory rate of 20/min, and SpO_2_ of 98% (O_2_ 5 L/min via mask). Because the cause of the severe subglottic stenosis was unclear and her symptoms were worsening day by day, we decided to perform emergency tracheostomy to prevent further worsening of her symptoms and a life-threatening condition.

The case was discussed in detail among otolaryngologists and anesthesiologists. Because we anticipated intubation to be impossible, emergency tracheostomy was planned. She was transferred to the operation room for emergency tracheostomy. Intraoperatively, the girl was monitored for oxygen saturation, end-tidal carbon dioxide, and non-invasive blood pressure, with electrocardiography performed as well.

We judged that it would be difficult to obtain the patient’s cooperation with only regional anesthesia. Emergent tracheostomy was therefore planned under general anesthesia. Sevoflurane was administered with sufficient titration to maintain spontaneous breathing. Anesthesia was maintained via the inhalation of air, oxygen, and sevoflurane. We considered that the use of a face mask instead of supraglottic airway devices during anesthesia might minimize the risk of tracheal injury or laryngospasm. The lungs were fully preoxygenated. The sevoflurane concentration was increased to 3%, at which point the patient was unresponsive.

The patient initially continued breathing spontaneously, but ventilation later became impossible because the otolaryngologists pulled her trachea. Before the oxygenation and hemodynamics became unstable, tracheostomy was successfully performed by the otolaryngologists. The patient was admitted to our pediatric intensive care unit (PICU) for postoperative airway management.

**Fig. 2:**
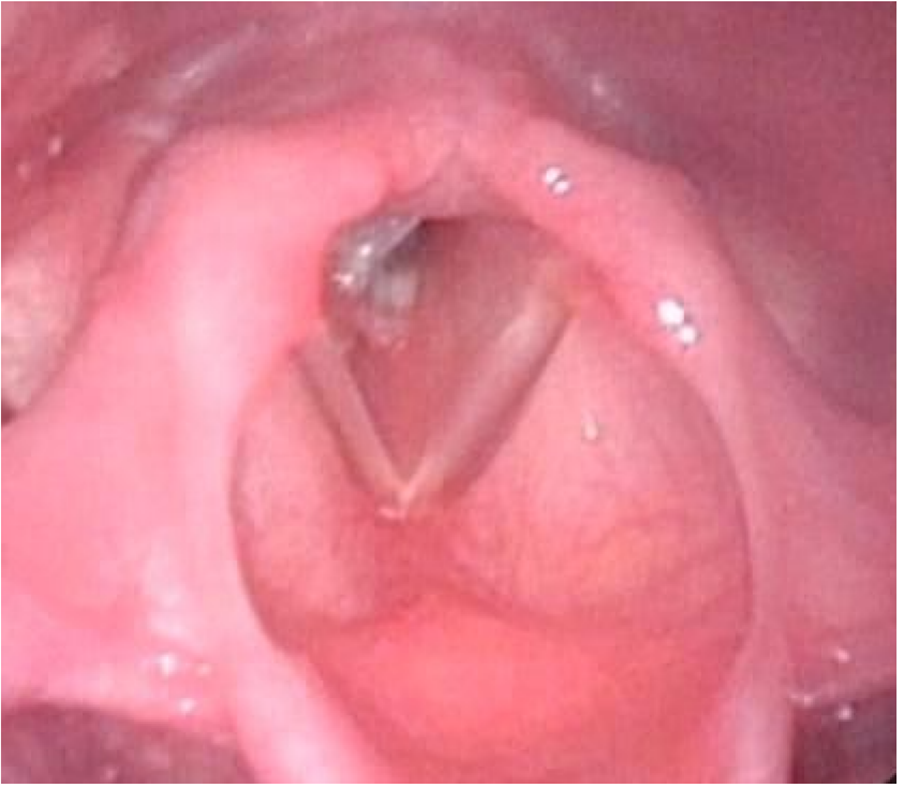
A laryngoscopic image that was thought to be severe edema at the subglottic stenosis on POD7

On postoperative day (POD) 3, because her airway had been stabilized by tracheostomy, she was discharged from the PICU. On POD 4, CT of the neck still showed narrowing of the subglottic portion. On POD 7, a laryngoscope under general anesthesia revealed severe edema at the subglottic stenosis (Fig. 2). The otolaryngologists considered that the cause of the subglottic stenosis might be edema due to cricoid fracture, given her medical history.

**Fig. 3:**
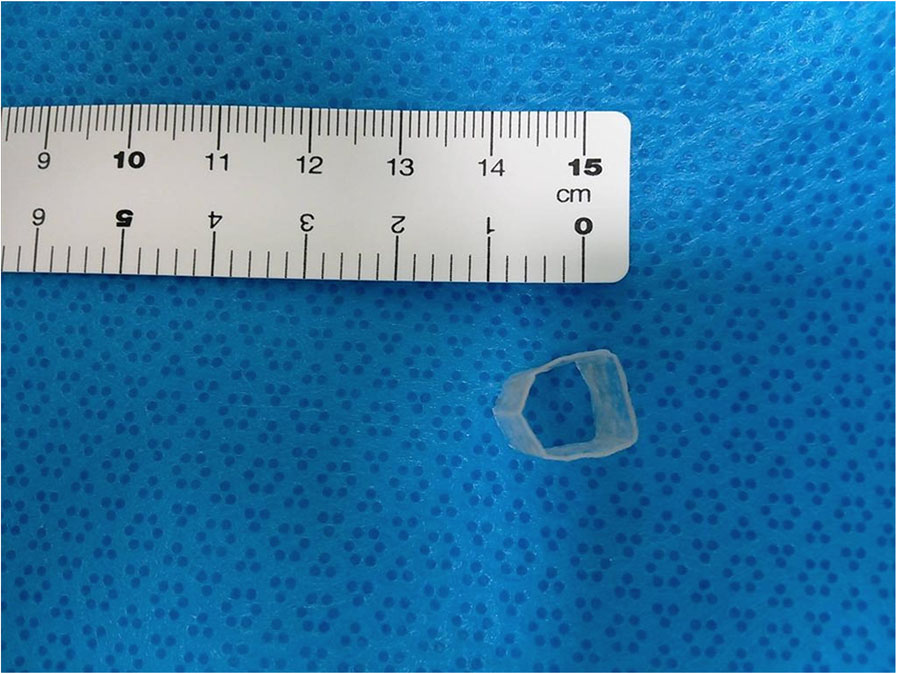
A circular plastic foreign body (cross-sectional diameter of 1.2 × 1.0 cm)

At five months after surgery, a laryngoscope was again inserted under general anesthesia, revealing large granulomas surrounding an object in the subglottic wall. Surprisingly, this object turned out to be a circular plastic foreign body (cross-sectional diameter of 1.2 × 1.0 cm) (Fig. 3). Surgery was performed to remove the object. On showing the foreign body to the patient and her parents, they did not remember her aspirating the object. She was discharged from our hospital and placed under home care after removal surgery.

## Discussion

Anesthetic management for airway emergency in such cases is always challenging. The patient’s subglottic diameter was much narrower than the average tracheal diameter measured at the subglottic level (for 4-year-olds, 9.2 mm in the transverse and 7.5 mm for the anteroposterior diameter) [2]. The anesthesia plan should therefore be reviewed with the surgeon in such cases. We had two options for securing the patient’s airway: tracheal intubation using video laryngoscopy or fiberoptic bronchoscopy, or surgical tracheostomy. Because we worried that attempted endotracheal intubation might worsen the mucosal edema, precipitating a near total obstruction, emergency tracheostomy was chosen to secure the patient’s airway. A collapsible extrathoracic airway narrows during inspiration and dilates during expiration because the transluminal pressure decreases during inspiration and increases during expiration [3]. We hypothesize that the subglottic airway became easily collapsed with the negative pressure developed during inspiration in the present case. Indeed, her airway was not rigid and collapsed easily during the surgical procedures. This patient with a highly collapsible airway should not have had general anesthesia induced without a plan for securing the airway.

The management of anesthesia and the airway for emergency tracheostomy in a child with severe subglottic stenosis has several accompanying issues. First, regional anesthesia is ideal for the anesthetic management of patients with subglottic stenosis if general anesthesia is not necessary. However, even awake techniques and regional anesthesia in the critical airway do not ensure safety [4]. Awake tracheostomy in this circumstance could not be guaranteed to be complication free and had the further disadvantage of risking laryngeal spasm. Sevoflurane was therefore administered with sufficient titration to maintain spontaneous breathing. Although we chose to use a gaseous induction of anesthesia rather than intravenous induction, some anesthesiologists have a strong preference for using a slowly increasing, incremental dose of target-controlled propofol while maintaining spontaneous ventilation. Propofol provides excellent anxiolysis that notably assists in the progress of anesthesia [3]. Because we worried that using a neuromuscular blocking agent might induce air trapping beyond stenosis during controlled ventilation, we decided to perform management under anesthesia while maintaining spontaneous breathing without a muscle relaxant. We expect that some aspects of our care might be considered controversial.

Second, the use of a face mask for airway management in severe subglottic stenosis during emergency tracheostomy is also certainly controversial. The use of a face mask can minimize tracheal injury during anesthesia, although this approach does not provide complete protection against aspiration. Supraglottic airway devices would be ineffective in such cases [5]. Of note, in one report of two cases of severe tracheal stenosis, the classic laryngeal mask airway (LMA) was ineffective in restoring ventilation, and both patients died [6]. Routine anesthesia induction and intubation in such cases can depress the patient’s auto-compensation, which can result in severe respiratory and cardiac consequences [7].

Third, when general anesthesia is necessary, the feasibility of mask ventilation under general anesthesia should be considered. If mask ventilation seems to be impossible, extracorporeal membrane oxygenation should be considered. In extreme cases, cardiopulmonary bypass under regional anesthesia has been used when performing tracheostomy. This approach allows for gas exchange and good surgical access for the tracheal operation and avoids serious hypoxia and carbon dioxide retention, which may result in cardiac arrest [8]. It should also be noted that tracheostomy in children with subglottic stenosis is challenging, even for experienced otolaryngologists [9].

As reported, strong teamwork, especially good communication between the medical care providers, is essential for ensuring a good patient outcome, preventing potentially avoidable complications, and thus reducing morbidity and mortality. Therefore, for the enhanced intraoperative management in our case, we felt that frequent communication between the anesthesiologists and otolaryngologists was very important [10].

Patients with acquired stenosis are typically diagnosed a few days or more after the initial injury. This case also showed that a healthy child can tolerate aspirated foreign bodies for a relatively long time [11]. We hypothesized that the traumatic event and aspirated foreign body had caused the marked subglottic stenosis in this case. It is important to obtain an accurate medical history in children who have specific respiratory problems, such as respiratory difficulty, noisy breathing, and frequent coughing with a poor response to therapies.

## Conclusions

Management of the airway and anesthesia in a child with severe subglottic stenosis should be selected according to each patient’s individual circumstances. In the presence of acute respiratory distress, definitive management should always take place in the operating room under anesthesia while maintaining spontaneous breathing without a muscle relaxant, and a skilled surgeon should be ready to perform an emergency tracheostomy. In the event of airway obstruction, preparation and team communication are paramount to avoid serious complications.

## Data Availability

The datasets are available from the corresponding author on reasonable request.

## Abbreviations

CT: Computed tomography
LMA: Laryngeal mask airway
